# Serine Phosphoacceptor Sites within the Core Protein of Hepatitis B Virus Contribute to Genome Replication Pleiotropically

**DOI:** 10.1371/journal.pone.0017202

**Published:** 2011-02-15

**Authors:** Eric B. Lewellyn, Daniel D. Loeb

**Affiliations:** McArdle Laboratory for Cancer Research, University of Wisconsin School of Medicine and Public Health, Madison, Wisconsin, United States of America; Yonsei University, Republic of Korea

## Abstract

The core protein of hepatitis B virus can be phosphorylated at serines 155, 162, and 170. The contribution of these serine residues to DNA synthesis was investigated. Core protein mutants were generated in which each serine was replaced with either alanine or aspartate. Aspartates can mimic constitutively phosphorylated serines while alanines can mimic constitutively dephosphorylated serines. The ability of these mutants to carry out each step of DNA synthesis was determined. Alanine substitutions decreased the efficiency of minus-strand DNA elongation, primer translocation, circularization, and plus-strand DNA elongation. Aspartate substitutions also reduced the efficiency of these steps, but the magnitude of the reduction was less. Our findings suggest that phosphorylated serines are required for multiple steps during DNA synthesis. It has been proposed that generation of mature DNA requires serine dephosphorylation. Our results suggest that completion of rcDNA synthesis requires phosphorylated serines.

## Introduction

Hepatitis B virus (HBV) is a widespread human pathogen that infects about 350 million people chronically [1]. A chronically infected individual has an elevated risk of developing liver cancer or liver failure. There are no therapeutic regimens that can eradicate a chronic infection [1]. A better understanding of the molecular biology of HBV replication is an important first step in the development of new therapeutic strategies. The current study focuses on understanding how the viral capsid participates in replication of viral nucleic acids.

HBV reverse transcribes a pregenomic RNA (pgRNA) to generate a double-stranded DNA genome with a relaxed-circular conformation (rcDNA). A diagram of these events is presented in Figure S1. First, the viral polymerase (P protein) associates with the pgRNA by binding a stem-loop, ε. The two are then co-encapsidated into a nascent capsid [2], [3], [4], [5], [6]. P protein, using a tyrosine at position 63 as a primer, initiates synthesis of minus-strand DNA [7], [8], [9]. Several unpaired nucleotides within ε are the template for synthesis of 3 to 4 nucleotides. The nascent minus strand switches template to an acceptor site that is near the 3′ end of the pgRNA [8], [10], [11], [12]. Minus-strand DNA is elongated [**(-) DNA elongation**] and RNase H activity of the P protein digests pgRNA with the exception of ∼17 nucleotides from the 5′ end. This RNA is the primer for initiation of synthesis of plus-strand DNA. The 3′ 11-nt of the primer contains the sequence called DR1. A second copy of this 11-nt sequence, called DR2, is located near the 5′ end of the minus-strand template. For the synthesis of rcDNA, the primer switches template to DR2, and (+) DNA synthesis initiates (**primer translocation**) [13], [14]. Synthesis of plus-strand DNA proceeds to the 5′ end of the template and then switches template to the 3′ end (**circularization**) [14]. Plus-strand DNA is elongated and rcDNA is generated [**(+) DNA elongation**]. In a minority of capsids, primer translocation does not occur and plus-strand DNA synthesis initiates from DR1 (**in situ priming**) to produce a duplex linear genome (dlDNA) [15].

The capsid contributes to genome replication through the carboxy-terminal domain (CTD) of the core protein. The CTD is an arginine-rich stretch of 34 amino acids, which is not required for capsid assembly [16], [17], [18]. Previous studies reported that the CTD plays a role in pgRNA encapsidation [16], [17], [19], [20], [21] and DNA synthesis [16], [17], [20], [21], [22]. Truncation of, or substitutions within, the CTD can cause a preferential reverse transcription of spliced pgRNAs [20], [21].

**Figure 1:**
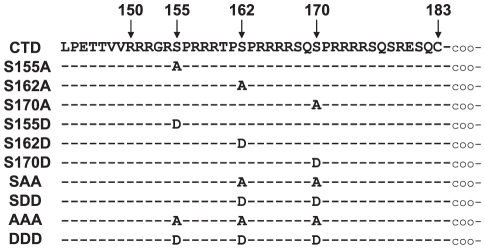
Sequence of wild type CTD and mutants. Positions of the three serine phosphoacceptor sites are shown. Below, each mutant is listed and differences from the wild type sequence are indicated.

The CTD can be phosphorylated at serines 155, 162, and 170 [19], [23], [24] (Fig. 1). Understanding how these serines contribute to reverse transcription and how phosphorylation affects that contribution is important to understand the mechanism for how the CTD functions. Replacing any of the three serines with alanine, aspartate, or glutamate reduces the amount of pgRNA that becomes encapsidated, but alanine substitutions cause the greatest reduction [19], [20], [22]. These findings suggest that phosphorylation is important for encapsidation because alanine can mimic dephosphorylated serine and aspartate or glutamate can mimic phosphorylated serine. Mutating these serines also causes reductions in rcDNA production [20], [22]. However, it is not clear which of the steps following encapsidation require these serines.

We show that serines 155, 162, and 170 contribute to DNA synthesis pleiotropically. Each serine makes an independent contribution to minus-strand DNA elongation, primer translocation, circularization and plus-strand DNA elongation. The alanine substitutions had a greater impact on genome replication that the corresponding aspartate substitution. Our findings suggest that phosphorylation may be important for both early and late steps of DNA synthesis.

## Results

The ability of core protein mutants to support the synthesis of DNA was analyzed by transfecting HepG2 cells with two plasmids. Plasmid EL100 expressed the pgRNA and P protein. pgRNA splicing was inactivated by a substitution within a 3′ splice site shared by most spliced HBV RNAs [25]. Plasmid EL43 expressed the WT core protein. Derivatives of EL43 were generated to express mutant core proteins in which the serines at positions 150, 162, or 170 were replaced with either an alanine or aspartate (Fig. 1). These mutants were analyzed to determine the contribution of each serine to reverse transcription of pgRNA and provide clues as to the role of phosphorylation of the CTD.

### Serines 155, 162, and 170 contribute to generation of rcDNA

**Figure 2:**
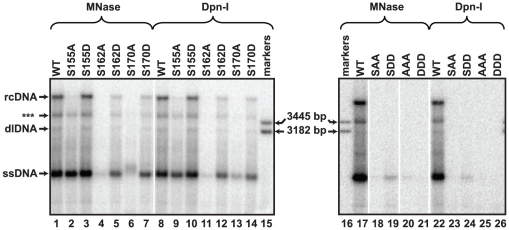
Southern blot analysis of capsid-derived DNA isolated from transfected cells. DNA was isolated from transfected cells by two different procedures. Cytoplasmic lysates were treated with MNase to digest unencapsidated nucleic acids and capsid DNA was subsequently isolated or total cytoplasmic DNA was isolated and then treated with the restriction enzyme DpnI, which digests transfected plasmid DNA. The positions of ssDNA, dlDNA, and rcDNA are indicated and the position of a linear, 3182 bp marker is also indicated. Each lane represents 1/3 of the HBV DNA from a 60 mm dish of transfected HepG2 cells. DNA was detected by hybridizing oligonucleotide probes 1833+, 1857+, 1876+, and 1995+ (Table S1). (***) indicates the discrete, incomplete rcDNA (ircDNA) molecule (See Fig. 3).

DNA synthesized by each mutant was analyzed by Southern blotting to determine the cumulative contribution of the serines to genome replication. rcDNA, dlDNA, and ssDNA have distinct mobilities and are found in characteristic proportions (referred at as “DNA profile”; e.g. Fig. 2; lane 1 for WT). Differences between each mutant and the wild type reference were apparent. Little, or no, rcDNA was detected for each mutant with single alanine substitutions (Fig. 2; lanes 2,4,6). Also, the level of full-length minus-strand DNA was less for each of the alanine substitutions relative to the level of the wild type reference (sum of signal between ssDNA and rcDNA; Fig. 2; lane 2,4,6). S162A accumulated the least amount of DNA, followed by S170A and S155A. When the DNA profile of each mutant with an alanine substitution was compared to the DNA profile of each corresponding aspartate substitution, the aspartate substitutions most resembled the wild-type DNA profile (Fig. 2; lanes 3,5,7). S155D had a DNA profile most similar to the wild type reference (Fig. 2; lane 3).

Mutants in which two or three serines were replaced with either alanines or aspartates also were analyzed (Fig. 2; lanes 18–21). DNA was not detected for SAA. Also, only a small amount of minus-strand DNA was produced by SDD. Replacing all three serines with either aspartates or alanines reduced DNA accumulation to undetectable levels (Fig. 2; lanes 20–21).

Previous studies reported that perturbations of the CTD lead to defects in pgRNA encapsidation [16], [17], [20], [21], [22], [24]. Therefore, some of the decreases we observed in DNA accumulation were likely due to defects in pgRNA encapsidation. In particular, Melegari et al. [22] reported that S162A leads to a profound encapsidation defect, S170P leads to a less profound decrease and S155A does not affect encapsidation [22]. We found that aspartate substitutions affected encapsidation less than alanine substitutions; S162D was the only aspartate substitution that encapsidated less pgRNA than the WT reference (Fig. S2). However, these decreases in pgRNA encapsidation did not account for the reductions in rcDNA accumulation that we observed. Therefore, we considered whether other steps in the synthesis of rcDNA were affected.

### Decreased accumulation of DNA for variants is not due to increased sensitivity to nuclease treatment during its isolation

The CTD of duck hepatitis B virus (DHBV) also is phosphorylated at multiple serine and threonine residues [26]. Mutating some of these residues can cause mature forms of the genome (i.e. full-length and nearly full-length rcDNA) to become susceptible during isolation to digestion with micrococcal nuclease (MNase) while the DNA is in the capsid [27], [28]. We omitted MNase during the isolation of viral DNA to determine whether a similar phenomenon was occurring in our analysis. The restriction endonuclease DpnI was used to remove transfected plasmid present in each sample. Viral DNA is resistant to digestion with DpnI. We found the pattern of viral DNA was similar when MNase or DpnI was used during isolation for each mutant (Fig. 2; MNase vs DpnI). Therefore, decreases in DNA accumulation for the mutants were not due to increased susceptibility to MNase.

### Mutating serines accentuates accumulation of a discrete, incomplete rcDNA molecule

**Figure 3:**
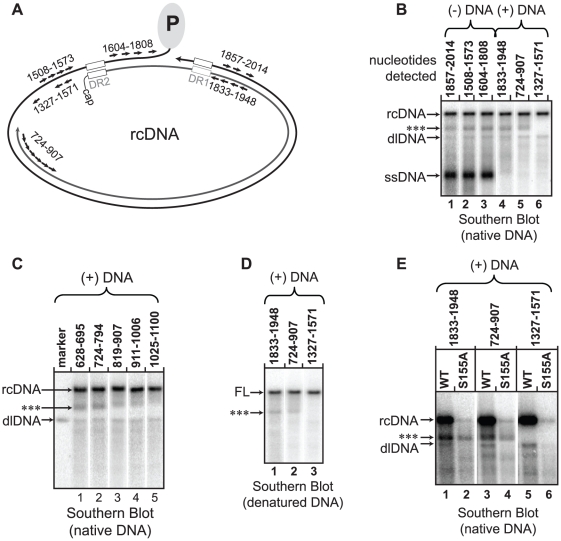
Evidence for an incomplete rcDNA form. Southern blots were hybridized with groups of radio-labeled oligonucleotide specific for either (+) DNA or (-) DNA and collectively detect the nucleotides that are indicated above each lane (Table S2 indicates the oligonucleotide probes were used). (**A**) The approximate positions of the oligonucleotide probes are shown relative to the rcDNA DNA. (**B**) A Southern blot of native DNA from the wild type reference is shown. Lanes 1-6 represents the same lane from a Southern blot that has been hybridized with different sets of oligonucleotides; old probes were removed from the membrane before new probes were used. The positions of rcDNA, dlDNA, and ssDNA are indicated. The unknown DNA species is indicated (***). (**C**) An additional Southern blot of native, WT DNA. The probes map the 3′ terminus of the unknown DNA species with greater specificity. (**D**) A Southern blot of denatured DNA. The position of full-length (+) DNA (FL) and an unknown DNA species that is less-than full length is indicated (***). (**E**) A native Southern blot of the variant S155A is shown along side the wild-type reference to indicate which probes do and do not detect the unknown DNA species in S155A (***). For all variants, 1/3 of the DNA from a transfected 60 mm dish was loaded in each lane.

Southern blotting revealed a discrete species of DNA that migrated between full-length rcDNA and dlDNA during agarose gel electrophoresis (Fig. 2; labeled as ***). Southern blotting was performed to identify the DNA in this band. Oligonucleotide probes specific for different regions of the genome were used to detect the DNA. The unidentified band was detected with probes for the 3′ and the 5′ ends of the minus strand (Fig. 3A; lanes 1-3). Probes specific for plus-strand DNA from nts 1883 to 1948 and nts 724 to 907 also detected the band (Fig. 3B; lanes 4,5). However, probes for the 3′ end of the plus strand did not detect this DNA form (Fig. 3B; lane 6). Probes that annealed between nts 794 and 1025 all detected the band, but the signal was less intense for probes closer to the 3′ end of plus-strand DNA (Fig. 3C; lanes 2–4). Probes 3′ of nt 1025 revealed a subtle smear that extended upward to rcDNA but the distinct band was not present (Fig. 3C; lane 5). Southern blots of heat-denatured DNA also had a discrete DNA species that migrated faster than full-length (+) DNA (Fig. 3D; ***). This band was detected with probes that were specific for the (+) DNA from nts 1833 to 907 and not probes for the 3′ end of (+) DNA (Fig. 3D). Together, these findings indicate that this band is a population of incomplete rcDNA genomes (ircDNA) that have heterogeneous 3′ termini between nts 794 and 1025.

The same probes that detected the ircDNA band in the wild type reference also detected a band in S155A, suggesting that S155A possesses the same ircDNA species as the wild type reference (Fig. 3E). The ircDNA of S155A remained prominent while the levels of rcDNA and dlDNA were diminished (Fig. 2). While the absolute level of ircDNA for S155A did not increase relative to the wild type reference, it did constitute a much greater proportion of total plus-strand DNA. An interpretation of this analysis is that the plus-strand of ircDNA is terminating or pausing at a discrete sites and that mutating serines within the CTD increases the proportion of plus strands that have terminated or paused at these sites.

### Strategy for quantitative analysis of individual steps of DNA synthesis

**Figure 4:**
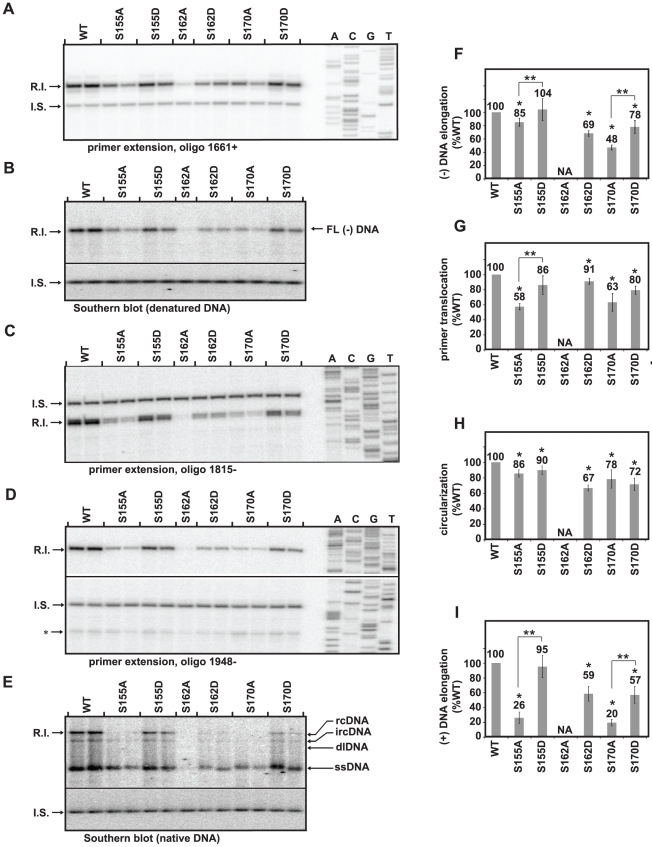
Analyses of (-) DNA elongation, primer translocation, circularization, and (+) DNA elongation. The position of the replicative intermediate band (R.I.) and internal standard band (I.S.) that were measured are indicated. (**A**) Primer extension with oligonucleotide 1661+ to detect (-) DNA that is, at least, 164 nt in length. (**B**) Southern blot with heat-denatured DNA to detect fully-elongated (-) DNA. (**C**) Primer extension with oligonucleotide 1815- to detect (+) DNA initiated from DR2. (**D**) Primer extension with oligonucleotide 1948- to detect circularized, DR2-initiated (+) DNAs. The position of in situ-primed (+) DNAs is indicated (*). (**E**) Southern blot with native DNA. Positions of ssDNA, dlDNA, ircDNA, and full-length rcDNA are indicated. Both native and denatured southern blots were probed with oligonucleotides 1816+, 1833+, 1857+, and 1876+. (**F–I**) Each histogram shows the efficiency of (-) DNA elongation, primer translocation, circularization, and (+) DNA elongation relative to the wild type reference, as calculated with equations in Fig. S3. S162A was not analyzed (NA) because DNA levels were below the limits of reliable quantitation due to accumulation of defects at earlier steps in replication. (*) indicates a significant difference between each variant and the wild type reference and (**) indicates a significant difference between the variants grouped by brackets (p<0.05). The standard deviation of each variant after normalization to the wild type reference is provided to give an impression of the sample variability, although non-parametric statistical tests were used for calculation of p-values.

We wanted to determine the efficiency of (-) DNA elongation, primer translocation, circularization, and (+) DNA elongation for each variant. Primer extension and Southern blotting were used to measure the relative amount of HBV DNA that had completed each of these steps. An internal standard DNA (I.S.) was used to directly compare measurements obtained by primer extension and Southern blotting. I.S. was added to each viral DNA prior to dividing the sample into 5 aliquots. DNA in each aliquot was analyzed using the assays depicted in Fig. 4A–E. Initiated (-) DNA was detected by primer extension using oligonucleotide 1661+ (Fig. S3A, Fig. 4A). Full-length (-) DNA was detected by Southern blotting in which samples were heat denatured (Fig. S3A, Fig. 4B). Plus-strand DNA initiated at DR2 was measured by primer extension using oligonucleotide 1815- (Fig. S3A, Fig. 4C). Circularized (+) DNA was detected by primer extension using oligonucleotide 1948- (Fig. S3A, Fig. 4D). Fully-elongated rcDNA was detected by Southern blotting of native DNA (Fig. S3A, Fig. 4E). The efficiencies of (-) DNA elongation, primer translocation, circularization, and (+) DNA elongation were then calculated using the formulas in Fig. S3B. An important feature of this strategy is the level of DNA for a given step is normalized to the level of DNA from the preceding step. For example, the level of primer translocation was normalized to the level of fully elongated (-) DNA. This strategy allows one to learn the efficiency of each step independent of the impact of the mutation on earlier steps.

### Serines 155, 162 and 170 contribute to (-) DNA elongation

Changing each of the serines to alanines decreased (-) DNA elongation (Fig. 4F) with S170A having the largest decrease at 48% the level of the wild type reference. S155A had a small decrease in the efficiency of (-) DNA elongation (85% the level of the wild type reference). S162A was not analyzed for this step or subsequent steps because of the magnitude of the cumulative defects from earlier steps. The mutants with aspartate substitutions elongated (-) DNA more efficiently relative to the corresponding alanine substitutions. S155D elongated (-) DNA as well as the wild type reference whereas S155A had reduced efficiency (Fig. 4F). Similarly, S170D elongated (-) DNA more efficiently than S170A (Fig. 4F), although S170D elongated (-) DNA less efficient than the wild type reference. S162D was also found to elongate (-) DNA less efficiently than the wild type reference.

### Serines 155, 162 and 170 contribute to primer translocation

We measured DNA that had undergone primer translocation, re-initiated at DR2, and synthesized at least 217 nucleotides (Fig. 4G). For ease of presentation, we will refer to these steps collectively as primer translocation. Each serine was found to contribute to this step. S155A and S170A had the lowest levels of primer translocation (58% and 63% the level of the wild type reference), but the three variants with aspartate substitutions also had decreased primer translocation efficiency (from 80% to 91% the level of the wild type reference). Comparison of the efficiency of primer translocation between variants S155A and S155D showed that variant S155A was less efficient.

### Alanine and Aspartate substitutions affect circularization similarly

All variants had lower levels of circularization than the wild type reference with levels ranging from 67% to 90% the level of the wild type reference (Fig. 4H). Unlike (-) DNA elongation and primer translocation, circularization was affected to a similar degree by aspartate and alanine substitutions.

### Serine 155, 162, and 170 contribute to plus-strand DNA elongation

All variants, except for S155D, had lower levels of (+) DNA elongation than the wild type reference (Fig. 4I). S155A and S170A had the largest decreases (26% and 20% the level of the WT, respectively). The aspartate substitutions were affected to lesser degree ranging from 57% to 95% of the WT reference. S155D was indistinguishable from WT and suggests serine phosphorylation at residue 155 could be necessary for (+) DNA elongation. An additional interpretation is that phosphorylation of all three serine residues is needed for (+) DNA elongation because the aspartate substitutions are better tolerated than the alanine substitutions.

## Discussion

We found that the three phosphoacceptor serines each contributed to DNA synthesis at multiple steps. The efficiency of plus-strand template switch steps and (-) and (+) DNA elongation processes were always decreased when serines were replaced with alanines (Fig. 4). In general, variants with alanine substitutions underwent each step less efficiently than variants with aspartate substitutions. The exception was circularization, which was equally affected by the alanine and aspartate substitutions. We found that these differences in efficiency were not because capsid-associated DNA had become progressively more susceptible to MNase digestion (Fig. 2). Finally, we found that an incomplete rcDNA, ircDNA, accumulates in both wild type and variant capsids (Fig. 3). However, the proportion of ircDNA relative to full-length rcDNA is increased in variants with an altered CTD.

Our findings indicate that the CTD contributes to DNA synthesis pleiotropically. Each of the serine phosphoacceptor sites is required for generation of rcDNA, although serine 162 is the most important and serine 155 is the least important of the three. This interprettion is consistent with an earlier report by Melegari et al. [22]. Kock et al. [20] also reported that no detectable full-length (-) DNA was generated when all three serines were replaced with glutamates, which is similar to our finding (Fig. 2 lane 22 and 26). Our study was carried out in the absence of spliced pgRNA. Thus, we conclude that the findings in the earlier reports (20, 22) were due to a defect in reverse transcription of the full-length genome rather than spliced genomes being preferentially encapsidated and thus limiting encapsidation of full-length genomes [20], [21].

We have shown that encapsidation of pgRNA, (-) DNA elongation, primer translocation, circularization, and (+) DNA elongation can be affected by mutations within the CTD despite these processes being mechanistically distinct. This observation must be considered when formulating models for how the CTD functions in genome replication. Our findings suggest that the CTD does not catalyze a specific template switch or bind to a specific nucleic acid structure as its primary mechanism. Our findings lead us to postulate that the CTD functions by interacting with nucleic acids non-specifically or interacting with the P protein. An intriguing idea, that is consistent with the data in this study, is that the CTD functions as a nucleic acid chaperone: a protein that facilitates conformational changes of nucleic acids [29]. The NC protein of retroviruses is thought to have nucleic acid chaperone activity. The NC protein of retroviruses plays a role in diverse processes such as primer annealing, template switching, and elongation through secondary structures in the RNA and DNA templates (for a review, [30]).

Our data suggests that the CTD is more active when it is phosphorylated. This was previously found to be the case for pgRNA encapsidation [19], [20], [22], [24], [31]. Our findings suggest that the subsequent steps of genome replication also benefit from a phosphorylated CTD. This speculation is based on the finding that mutants with aspartate substitutions are more like the wild type reference than alanine substitutions. Alanines can be functionally equivalent to dephosphorylated serines because both are neutral. Phosphorylated serines and aspartates have a negative charge so aspartates can be functionally equivalent to phosphorylated serines. This difference between variants with alanine and aspartate substitutions was observed for (-) DNA elongation, primer translocation, and (+) DNA elongation.

We characterized a DNA form that migrates between dlDNA and rcDNA in Southern blotting. The form was determined to be an rcDNA genome with an incomplete (+) DNA (ircDNA). It has long been appreciated that HBV DNA virions can contain an incompletely elongated (+) DNA that is typically less than 1800 nts in length [14], [32], [33]. We found that the plus strand of ircDNA was 2200 to 2400 nts long. We would expect that capsids containing ircDNA to be secreted as virions. Perhaps the biological role of ircDNA is to limit the production of intracellular amplification of cccDNA. There is evidence that only full-length rcDNA can be converted to cccDNA [34]. Arresting genomes in a state in which they can be secreted but cannot be converted to cccDNA, may be a means by which the virus limits the production of cccDNA.

ircDNA was less abundant than full-length rcDNA in the wild type reference. Interestingly, variants with serine to alanine substitutions generated more ircDNA than full-length rcDNA (Fig. 2, 3). These same variants were found to have less elongation of (+) DNA (Fig. 4I). Therefore, it is likely that part or all of the decrease in elongation of (+) DNA was due to a decreased ability to elongate the (+) DNA beyond ircDNA. A better understanding of why ircDNA forms should yield valuable insights into how the CTD functions. One possibility is that a structure within the (-) DNA template impedes the P protein during (+) DNA elongation. Consequently, elongation through ircDNA represents a rate-limiting step during elongation. If this scenario is accurate, our data suggests the CTD facilitates DNA polymerization through this putative structure.

The interpretation that phosphorylated CTD appears to be more active than the dephosphorylated form raises important questions about the role of dephosphorylation during maturation of HBV genomes. DHBV capsids in secreted virions and intracellular capsids with mature genomes are hypophosphorylated [26], [35]. Intracellular DHBV capsids containing immature genomes are hyperphosphorylated [26]. However, it is not known whether a conversion from hyperphosphorylated core to hypophosphorylated core also occurs prior to virion secretion in HBV. Future studies to investigate the phosphorylation state of HBV capsids at different stages of genome maturation are required to resolve this issue.

Our study suggests that dephosphorylation of capsids is not required for completion of (+) DNA and are inconsistent with the hypothesis that completion of the (+) DNA requires dephosphorylation of the CTD [36]. This earlier hypothesis was motivated by several observations. Capsid substitutions E113A and E117A could partially reverse a replication defect caused by replacing core residues 172–183 with two glycines (173GG) [36]. Residues E113 and E117 CTD and residues 172–183 are thought to be disposed to the capsid interior [36]. This finding, along with the earlier report that HBV replication cannot tolerate CTD truncations beyond residue 173 [21], inspired the charge balance model for HBV replication, which postulates that the overall positive charge of the capsid interior is important for genome replication because it counteracts the negative chare of encapsidated nucleic acids [21]. Based on this model, it was further hypothesized that dephosphorylation during (+) DNA synthesis could serve to maintain the charge balance as the synthesis of (+) DNA adds additional negative charges to the capsid interior. In contrast to these predictions, our data suggest that dephosphorylation at residues 155 and 170 (and possibly 162) is detrimental to (+) DNA elongation. A more likely explanation for the earlier observation that E113A and E117A can restore the replication defect of 173GG is that these residues are contributing to pgRNA encapsidation, a step for which the net charge of the capsid lumen does appear to be important [21], [29], [37]. If core is dephosphorylated prior to secretion, our data suggest that (+) DNA would not be completed, as is observed in secreted virions [33].

## Materials and Methods

### Molecular Clones

All HBV expression plasmids were derived from the sequence found at GenBank accession number V01460 (subtype ayw, genotype D). Nucleotide position 1 was designated to be the C of the unique EcoR1 site (GAATTC) in the positive polarity.

The wild-type (WT) core protein was expressed from plasmid EL43, which is a derivative of previously-described plasmid LJ96 [38]. EL43 contains a 1.04-mer of HBV sequence starting at nucleotide 1858 and ending at nucleotide 1986. Nucleotide numbering goes from 1858 to 3182 and restarts at 1. This sequence excludes part of ε and prevents encapsidation of the expressed mRNA. Substitution T2342A creates a premature stop codon in codon 13 of P protein, T154C disrupts the S protein start site, and C169A creates a premature stop codon in all envelope proteins. All core protein mutants are derived from EL43. P protein and pgRNA were expressed from plasmid EL100, which is a derivative of previously-described LJ144 [25] and differs by the addition of a premature stop codon in the C gene by changing nucleotides at position 1908 from GACC to TAAG, and has an inactivated 3′ splice acceptor site due to alteration of the nucleotides at position 484 from CAGG to GTCC. All variants were generated by oligonucleotide mutagenesis via PCR or cloning fragments from other plasmids. All molecular clones were confirmed by sequencing across the entire restriction fragment insert. Plasmid LJ200 [39] was used as an internal standard for DNA primer extension and Southern blot analyses. LJ200 was digested with Hph-I (New England Biolabs, Ipswitch, MA) to generate fragments of the appropriate size for the assays.

### Cell Culture and transfection

The human hepatoma cell line HepG2 [40] was used in all analyses. Cell culturing and transfection via calcium phosphate precipitation were carried out as previously described [38], [39], except 9 µg of each HBV plasmid were transfected with 1 µg of an expression plasmid for green fluorescent protein for a total of 19 µg DNA per dish. Cells were harvested on day 3 for analysis of pgRNA encapsidation and day 5 for analysis of all DNA synthesis steps.

### Isolation of viral replicative intermediates

For all analyses, cell cultures were washed with 2 mM HEPES, 150 mM NaCl, 0.5 mM EGTA [pH 7.45] and then frozen at −70°C. Later, cells were thawed to room temperature and lysed with 0.2% NP40, 50 mM Tris-HCL, and 1 mM EDTA [pH 8.0] for 10 minutes. Nuclei were removed by centrifugation at 16,000× g for 4 minutes at 4°C as described [38], [39]. For isolation of encapsidated viral nucleic acids, lysates were adjusted to 2 mM CaCl_2_ then incubated with 44 units of micrococcal nuclease (Worthington, Lakewood, NJ) to digest transfected plasmid DNA and unencapsidated HBV RNA. After two hours, these reactions were supplemented with EDTA to 10 mM, SDS to 0.4% and Pronase (Roche, Basel, Switzerland) to 400 µg/ml for 2 hours at 37°C. Nucleic acids were then extracted with a phenol/chloroform mixture and precipitated with ethanol. For DNA preps, 1 µg of RNase A was added to each sample. For isolation of DNA in the absence of MNase, all steps were the same except MNase and CaCl_2_ was omitted. To digest the transfected plasmid, 10 units of restriction endonuclease, DpnI (New England Biolabs, Ipswitch, MA), was added to each sample for two hours at 37°C.

### Southern Blot Analyses

Native or heat denatured (95° for 5 minutes) viral DNA was electrophoresed through a 1.25% agarose gel in TBE buffer (2.5 mM EDTA, 90 mM TRIS, and 90 mM Borate), transferred by capillary action to Hybond-N membrane (GE healthcare, Piscataway, NJ), and cross-linked to the membrane with ultraviolet light. Membranes were incubated in Church hybridization solution (5 mM EDTA, 1% BSA, 0.25 M Na_2_HPO_4_, 7% SDS [pH 7.2]) for 1 hour at 42°C prior to addition of radio-labeled, oligonucleotide DNA probes. The membrane and probes were hybridized at 42°C overnight in Church hybridization solution. Membranes were then washed with Church wash solution (1% SDS, 20 mM Na_2_HPO_4_, 1 mM EDTA). Five washes were carried out at room temperature and followed by one wash at 42°C. Autoradiographic images of the membrane were obtained using a GE Healthcare Typhoon 8600 phosphorimager and quantitated using ImageQuant 5.2 software (GE Healthcare, Buckinghampshire, UK). Detection of HBV DNA was achieved by using radiolabeled oligonucleotide DNA probes. For Fig. 2, probes 1833+, 1857+, 1876+, and 1995+ were used (Table S1). For Fig. 3, the probes used for detecting the indicated regions of the genome are listed in Table S2. In Fig. 4, HBV DNA was detected using probes 1816+, 1833+, 1857+, and 1876+ (Table S1). Detection of the internal standard DNA (I.S.) in Fig. 4 was achieved by using radio-labeled oligonucleotide probes b1, b2, b3, and b4 (Table S1) that were specific for the non-HBV region of plasmid LJ200. All oligonucleotides were 5′-end labeled with [γ-^32^P] ATP using T4 polynucleotide kinase (New England Biolabs, Ipswich, MA) as described [25].

For Southern blots in Fig. 4, the HBV DNA was detected first, the probes were removed from the membranes, and the I.S. DNA was detected subsequently. To remove oligonucleotide probes between hybridizations, membranes were incubated in water for 30′ at 70°C and removal was demonstrated by obtaining an autoradiographic image of each membrane. Multiple masses of a plasmid DNA that contained both HBV and I.S. sequence were included on each blot in lanes adjacent to the HBV samples. The plasmid DNA was used to determine the mass of HBV DNA and I.S. DNA in each sample so that the mass of HBV DNA could be normalized to the mass of plasmid DNA. By comparing calculated masses, we have normalized for differences in detection efficiency between the I.S.- and HBV-specific probes.

### Primer Extension Analysis

All oligonucleotide primers were 5′-end labeled with [γ-^32^P] ATP using T4 polynucleotide kinase (New England Biolabs, Ipswich, MA) as described [25]. The approximate annealing positions of oligonucleotide primers are indicated in figure diagrams. The sequences of specific oligonucleotide DNA primers are given in Table S1.

In the analyses in Figure 4, 2 ng of I.S. DNA and 1 µg RNase A (Sigma-Aldrich, St. Louis, MO) were added to each viral DNA sample. Samples were incubated for 2 hrs at 37°C, precipitated with ethanol, and resuspended in H_2_O. The rehydrated sample was then split into five equal portions and primer extension analysis was performed on three aliquots using three oligonucleotide DNA primers, one per reaction, with Vent exo- polymerase (New England Biolabs, Ipswich, MA) as described [39]. Products of the primer extension reactions were electrophoresed through 6% polyacrylamide gels containing 7.6 M urea. Gels were dried and autoradiography was performed using a phosphorimager as described for DNA analysis by Southern blotting (Fig. 4A, C, and D). The remaining two aliquots of the sample were analyzed by Southern blotting; one with heat-denatured samples and the other with native DNA (Fig. 4B and E). For analysis of pgRNA encapsidation using primer extension (Fig. S2) we conducted reverse transcription primer extension as described [29].

### Statistical Analysis

For quantitative analysis in Fig. 4, each mutant was analyzed in three or more independent experiments, each containing at least two mutant samples and two wild type reference samples. An experiment consisted of samples that were transfected and analyzed together. Each mutant was compared to the wild type and, when indicated, to other variants. Analyzing samples from multiple experiments was accomplished using a permutation test, as described by Lehmann, *Example 6* [41]. Sample data from (-) DNA elongation experiments and P-values calculated from those data are provided in Table S3, as an example. P-values were calculated using the program Mstat 5.4.4 (Provided by Dr. Norman Drinkwater, University of Wisconsin). P<0.05 was considered statistically significant. The histograms in Fig. 4 show the level of each variant normalized to the wild type, but for the purpose of statistical analysis, samples were not normalized to the wild type reference.

## Supporting Information

Figure S1 **Synthesis of double-stranded DNA from single-stranded pgRNA.** **(A)** The pgRNA is greater than genome length and has 5′ and 3′ copies of the 11-nucleotide direct repeat DR1 and the stem loop ε. The relative position of the other direct repeat, DR2, is also indicated. pgRNA is translated to make the core protein and P protein. P protein associates with ε and the combination of the two comprises the encapsidation signal. **(B)** Minus-strand DNA synthesis initiates within the bulge of ε. Tyrosine 63 of P protein is the primer for the first 3-4 nucleotides and the bulge of ε is the template. The (-) DNA then switches template to an acceptor site within the 3′ copy of DR1. **(C)** **(-) DNA elongation.** Minus-strand DNA elongation and concomitant RNAse H digestion of the pgRNA are catalyzed by the P protein, resulting in a full-length, minus-strand DNA. The 5′-end of the pgRNA is not digested. **(D)** **Primer translocation**. The pgRNA remnant undergoes a template switch from DR1 to DR2 and primes (+) DNA synthesis from DR2. **(E) Circularization.** After synthesizing to the 5′ end of the minus-strand DNA template, the 3′ end of the plus strand switches templates to use the 3′ end of (-) DNA. **(F) (+) DNA elongation**. Once annealed to 3′r, the (+) DNA synthesis resumes, ultimately yielding rcDNA. **(H) In situ priming.** In a minority of cases, the RNA primer does not undergo primer translocation and primes from DR1. **(I)** When the products of in situ priming are elongated fully, a dlDNA genome is produced.
(EPS)

Figure S2 **The encapsidation of the 5′ end of pgRNA. (A)** Position of oligonucleotide 1948- used to measure pgRNA by primer extension. **(B)** Histogram showing the efficiency of encapsidation of the 5′ end of pgRNA relative to the WT reference. **(C)** Equation used to calculate the efficiency of encapsidation of the 5′ end of the pgRNA. **(D)** and **(E)** are images of gels containing the total and encapsidated pgRNA, respectively. The pgRNA is the band of interest and the ref. RNA is another pgRNA isolated from a dish containing WT core and a pgRNA with a 6 nt insertion to differentiate it from the WT [29]. Cytoplasmic lysates from the pgRNA of interest and the ref. RNA were mixed prior to isolation. Each sample was then divided into MNase-treated and untreated fractions. The MNase-treated fraction contained encapsidated RNA, the untreated contained total. Each sample was analyzed in duplicate.
(EPS)

Figure S3 **Formula to calculate the relative efficiency of (-) DNA elongation, primer translocation, circularization, and (+) DNA elongation. (A)** Position of all oligonucleotide primers and Southern blot hybridization probes used to detect DNA at various stages of maturation. Primer extension analysis with oligonucleotide 1661+ detects all replicative intermediates that have undergone minus-strand DNA initiation, the template switch and synthesizes of at least 164 nt of (-) DNA. A Southern blot with denatured DNA was used to detect all replicative intermediates with a fully-elongated (-) DNA; the approximate annealing position of the four tandem probes is indicated (see Materials and Methods for details). Primer extension with oligonucleotide 1815- to detect all (+) DNA molecules initiated from DR2 and had synthesized, at least, 217 nt. Primer extension with oligonucleotide 1948- to detect all (+) DNA molecules that were initiated from DR2, circularized, and synthesized, at least, 250 nt. Southern blotting of native DNA was used to detect fully-elongated rcDNA molecules using the indicated probes. **(B)** Equations for calculation of (-) DNA elongation, primer translocation, circularization, and (+) DNA elongation are shown.
(EPS)

Table S1 **Oligonucleotides used as hybridization probes to detect minus- or plus-strand DNA in Southern blot analysis.**
(DOC)

Table S2 **Nucleotides detected by combinations of oligonucleotide probes for Southern blotting.**
(DOC)

Table S3 **Sample data set and P-values calculated by permutation test** [**41**] **.**
(DOC)
