# Supplementary material for: Serine Phosphoacceptor Sites within the Core Protein of Hepatitis B Virus Contribute to Genome Replication Pleiotropically
Source: PLoS One. 2011 Feb 15;6(2):e17202. doi: 10.1371/journal.pone.0017202 (PMC3039676; doi:10.1371/journal.pone.0017202)
Supplement: Table S1 — Oligonucleotides used as hybridization probes to detect minus- or plus-strand DNA in Southern blot analysis. (DOC) [file pone.0017202.s004.doc]

**Table S1.** Oligonucleotides used as hybridization probes to detect minus- or plus-strand DNA in Southern blot analysis

| **Oligo^a^** | **Sequence (5’ to 3’)** |
| --- | --- |
| 1816+ | AACTTTTTCACCTCTGC |
| 1833+ | CTAATCATCTCTTGTTCATGTC |
| 1857+ | ACTGTTCAAGCCTCCAAGCT |
| 1876+ | TGTGCCTTGGGTGGCTTTGG |
| 1995+ | ACCGCCTCAGCTCTGTATCG |
| 1508+ | CGACCGACCACGGG |
| 1522+ | GCGCACCTCTCTTTACGC |
| 1540+ | GGACTCCCCGTCTGTG |
| 1556+ | CCTTCTCATCTGCCGGAC |
| 1604+ | GCATGGAGACCACCGTGAA |
| 1661+ | CTCTTGGACTCTCAGCAATGTCAAC |
| 1767+ | TGTACTAGGAGGCTGTAGGCAT |
| 1794+ | GGTCTGCGCACCAGC |
| 1815- | GCATGGTGCTGGTGC |
| 1859- | AGGACATGAACAAGAGATGATTAG |
| 1878- | GCTTGGAGGCTTGAAC |
| 1909- | TAGATGTCCATGCCC |
| 1948- | GAGAGTAACTCCACAG |
| 650- | GGCCCACTCCCATAGGAATTTT |
| 668- | CCAGGAGAAACGGGCTGA |
| 695- | CTGAACAAATGGCACTAGTAAACTGAG |
| 750- | ACCACATCATCCATATAACTGAAAGCC |
| 770- | GTACAGACTTGGCCCCCAAT |
| 794- | CGGTAAAAAGGGACTCAAGATGCT |
| 850- | CTTTGTTTTGTTAGGGTTTAAATGTATACCC |
| 880- | ACCCATAAAATTTAGAGAGTAACCCCATCT |
| 907- | CAAGGACCCATAACATCCAATGACATA |
| 950- | TTCTAAAACATTCTTTGATTTTTTGTATGATGTGTTCTT |
| 979- | CCAATCAATAGGCCTGTTAATAGGAAGTT |
| 1006- | AAGACCCACAATTCGTTGACATACTTT |
| 1050- | AACGCAGGATAACCACATTGTGTAA |
| 1080- | TTAGATTGAATACATGCATACAAAGGCATC |
| 1100- | GCGAGAAAGTGAAAGCCTGC |
| 1350- | GATAGGACAACAGAGTTATCAGTC |
| 1500- | AGACGGAGAAGGGGACGAGAG |
| 1571- | CCGGCAGATGAGAAGGCAC |
| b1 | GAGCCTATGGAAAAACGCCA |
| b2 | GCAACGCGGCCTTTTTAC |
| b3 | AGCGTCGATTTTTGTGATGCT |
| b4 | TTCGCCACCTCTGACTTG |

^a^. Oligonucleotides with positive polarity (+) anneal to (-)

HBV DNA. Oligonucleotides with minus polarity (-) anneal to plus-

strand HBV DNA. Oligonucleotides b1-b4 are specific for the non-

HBV region of the I.S.
