# Supplementary material for: Serine Phosphoacceptor Sites within the Core Protein of Hepatitis B Virus Contribute to Genome Replication Pleiotropically
Source: PLoS One. 2011 Feb 15;6(2):e17202. doi: 10.1371/journal.pone.0017202 (PMC3039676; doi:10.1371/journal.pone.0017202)
Supplement: Table S2 — Nucleotides detected by combinations of oligonucleotide probes for Southern blotting. (DOC) [file pone.0017202.s005.doc]

**Table S2.** Nucleotides detected by combinations of oligonucleotide

probes for Southern blotting.

| **Nucleotides**  **Detected** | **Strand**  **Detected** | **Oligonucleotide**  **Probes** |
| --- | --- | --- |
| 1857-2014 | (-) DNA | 1857+, 1876+, 1995+, |
| 1508-1573 | (-) DNA | 1508+, 1522+, 1540+, 1556+ |
| 1604-1808 | (-) DNA | 1604+, 1661+, 1767+, 1794+ |
| 1833-1948 | (+) DNA | 1859-, 1878-, 1909-, 1948- |
| 628-695 | (+) DNA | 650-, 668-, 695- |
| 724-794 | (+) DNA | 750-, 770-, 794- |
| 724-907 | (+) DNA | 750-, 770-, 794-, 850-, 880-, 907- |
| 819-907 | (+) DNA | 850-, 880-, 907- |
| 911-1006 | (+) DNA | 950-, 979-, 1006- |
| 1025-1100 | (+) DNA | 1050-, 1080-, 1100-, |
| 1327-1571 | (+) DNA | 1350-, 1500-, 1571- |
