# Supplementary material for: Serine Phosphoacceptor Sites within the Core Protein of Hepatitis B Virus Contribute to Genome Replication Pleiotropically
Source: PLoS One. 2011 Feb 15;6(2):e17202. doi: 10.1371/journal.pone.0017202 (PMC3039676; doi:10.1371/journal.pone.0017202)
Supplement: Table S3 — Sample data set and P-values calculated by permutation test [41] . (DOC) [file pone.0017202.s006.doc]

Table S3. Sample data set and P-values calculated by permutation test [41].

| **Experiment**  **(Block Number)** | **(-) DNA elongation efficiency^a^** | | |
| --- | --- | --- | --- |
|  | **WT** | **S170A** | **S170D** |
| 1 | 38.8, 40.1 | 18.3, 19.5 | 29.1, 26.4 |
| 2 | 35.2, 31.8 | 15.4, 15.7 | 26.5, 24.1 |
| 3 | 24.5, 24.0 | 13, 10.4 | 20.5, 22.7 |
| P-value | | 0.0093**^b^** | 0.0093**^b^** |
|  |  | 0.0093**^c^**. | |

a. (-) DNA elongation efficiency = [FL (-) DNA/I.S.]/[initiated (-) DNA/I.S.], (Fig. 4A, equations Fig. S3)

b. P-value when compared to the WT reference

c. P-value when the two variants are compared to one another
